# A Reporter Screen in a Human Haploid Cell Line Identifies CYLD as a Constitutive Inhibitor of NF-κB

**DOI:** 10.1371/journal.pone.0070339

**Published:** 2013-07-08

**Authors:** Clarissa C. Lee, Jan E. Carette, Thijn R. Brummelkamp, Hidde L. Ploegh

**Affiliations:** 1 Whitehead Institute for Biomedical Research, Cambridge, Massachusetts, United States of America; 2 Department of Biology, Massachusetts Institute of Technology, Cambridge, Massachusetts, United States of America; Tufts School of Medicine, United States of America

## Abstract

The development of forward genetic screens in human haploid cells has the potential to transform our understanding of the genetic basis of cellular processes unique to man. So far, this approach has been limited mostly to the identification of genes that mediate cell death in response to a lethal agent, likely due to the ease with which this phenotype can be observed. Here, we perform the first reporter screen in the near-haploid KBM7 cell line to identify constitutive inhibitors of NF-κB. CYLD was the only currently known negative regulator of NF-κB to be identified, thus uniquely distinguishing this gene. Also identified were three genes with no previous known connection to NF-κB. Our results demonstrate that reporter screens in haploid human cells can be applied to investigate the many complex signaling pathways that converge upon transcription factors.

## Introduction

Forward genetic screens are a powerful means to decipher a biological process without any prior knowledge or assumptions. Typically such screens are performed in yeast, *Drosophila, Caenorhabditis elegans* and other genetic model organisms to identify new gene functions. Application of this method to human cultured cells allows the dissection of pathways that are dissimilar or even absent in other model organisms. It may also enable the discovery of novel drug targets to treat disease. Genetic screens in human cells have been limited by the difficulties inherent in revealing recessive phenotypes in diploid cells. While RNAi screens have been an important advance, they are complicated by off-target effects and often do not completely eliminate the relevant gene product. The recent isolation of human cells lines that are nearly or completely haploid (KBM7 and HAP1, respectively) has revolutionized human forward genetic screens and led to the identification of numerous human host factors required for infection by pathogens and intoxication by bacterial toxins [1–7].

The majority of human haploid screens reported to date have involved the selection of mutants that are resistant to an agent that is lethal to wild-type cells. The one exception is a recent screen that used fluorescence activated-cell sorting (FACS) to identify genes involved in MHC (major histocompatibility complex) class I antigen presentation by sorting for mutants that were defective in surface expression of MHC-1 [8]. We sought to further expand the types of biological pathways that can be studied using human haploid genetic screens by using a transcriptional reporter in conjunction with selection for a lethal phenotype.

Transcription factors often lie at the terminus of complex signaling pathways and control gene transcription programs that regulate diverse processes, ranging from proliferation, differentiation, apoptosis, immune response, to metabolism. Given the importance of transcription factors in facilitating vital aspects of cell biology, mutations in -or aberrant regulation of-transcription factors have been associated with human disease [9,10]. The identification of inhibitors or activators of transcription factors will therefore not only illuminate the signaling pathways that regulate them, but could also identify targets that may prove to be better drug targets than transcription factors themselves, or whose inhibition may provide a more selective therapeutic effect.

We chose to screen for inhibitors of NF-κB, a family of transcription factors that in mammals plays a central role in regulating immune responses, development, cell proliferation, and survival [11]. The NF-κB family consists of five members: RelA/p65, RelB, c-Rel, NF-κB1 (p50 and its precursor p105) and NF-κB2 (p52 and its precursor p100). They form dimers and are normally kept inactive in the cytoplasm. Activation of a wide variety of receptors, including antigen receptors, pattern-recognition receptors and cytokine receptors leads to translocation of NF-κB dimers into the nucleus. Here the dimers bind to DNA κB sites in promoters and enhancers of target genes. Activation of NF-κB needs to be tightly controlled and rapidly curtailed following the initial stimulus to prevent uncontrolled tissue damage and/or disease.

Here we performed the first reporter screen in KBM7 cells to identify constitutive inhibitors of NF-κB. The identification of CYLD, a known negative regulator of NF-κB, demonstrates the utility of using human haploid cells to dissect a variety of biological processes.

## Results

**Figure 1:**
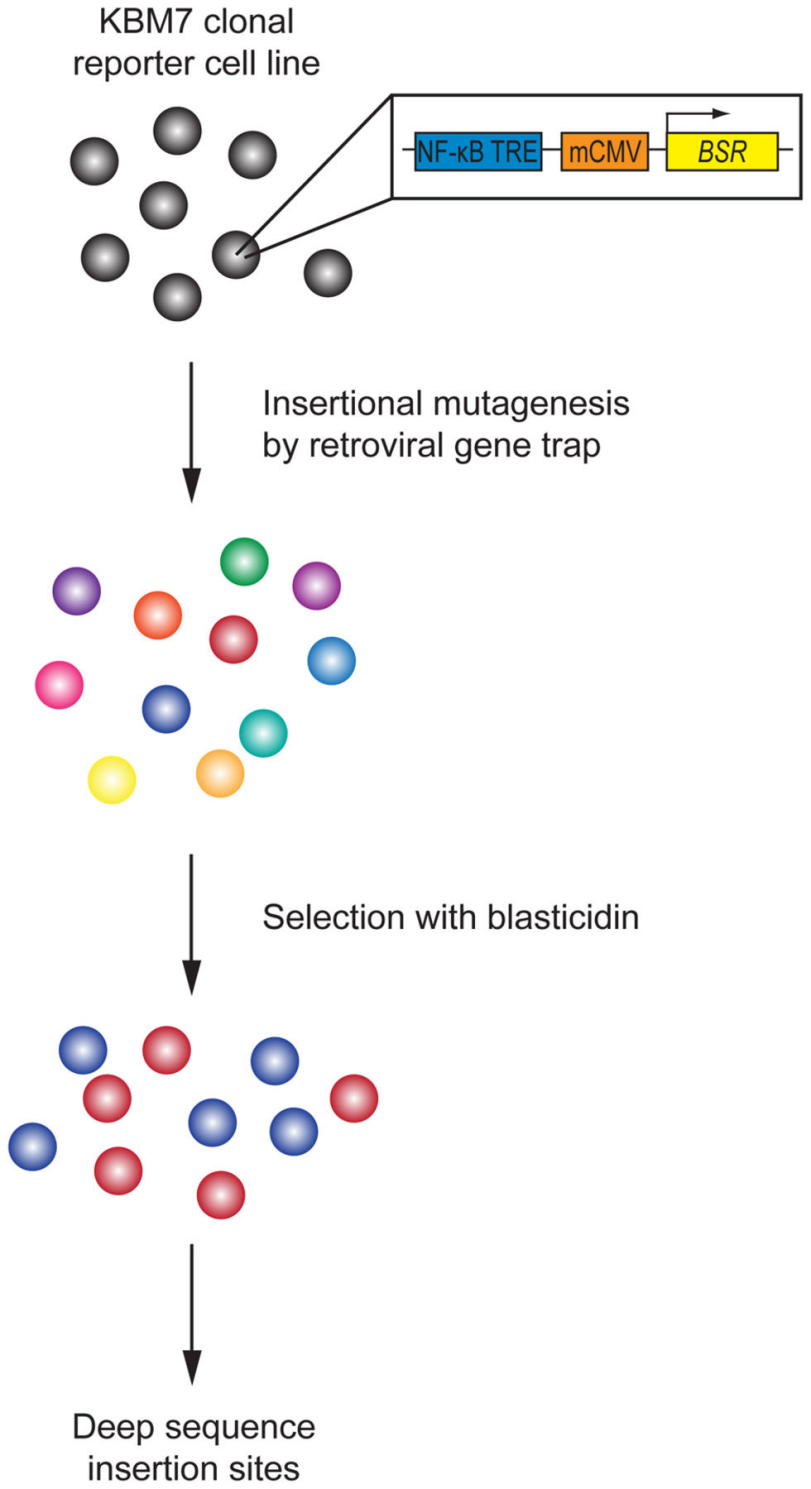
NF-κB reporter haploid genetic screen. KBM7 cells were transduced with a reporter containing a NF-κB transcriptional response element (TRE) and a minimum CMV (mCMV) promoter upstream of the blasticidin S resistance gene (*BSR*) from *Bacillus cereus*. A clonal reporter cell line was mutagenized by infection with a gene-trap virus. The resulting cells were treated with blasticidin. Survivors were expanded and DNA was extracted. DNA sequences flanking gene-trap insertion sites were amplified and sequenced in parallel.

**Figure 2:**
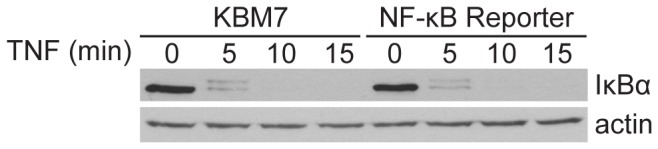
NF-κB activity is regulated in KBM7 cells. KBM7 and NF-κB reporter cells were stimulated with 10 ng/mL TNF for the times indicated. Lysates were analyzed by immunoblot with anti-IκBα. After stripping, the membrane was reprobed with anti-actin antibodies to provide a loading control. Data are representative of two independent experiments.

**Figure 3:**
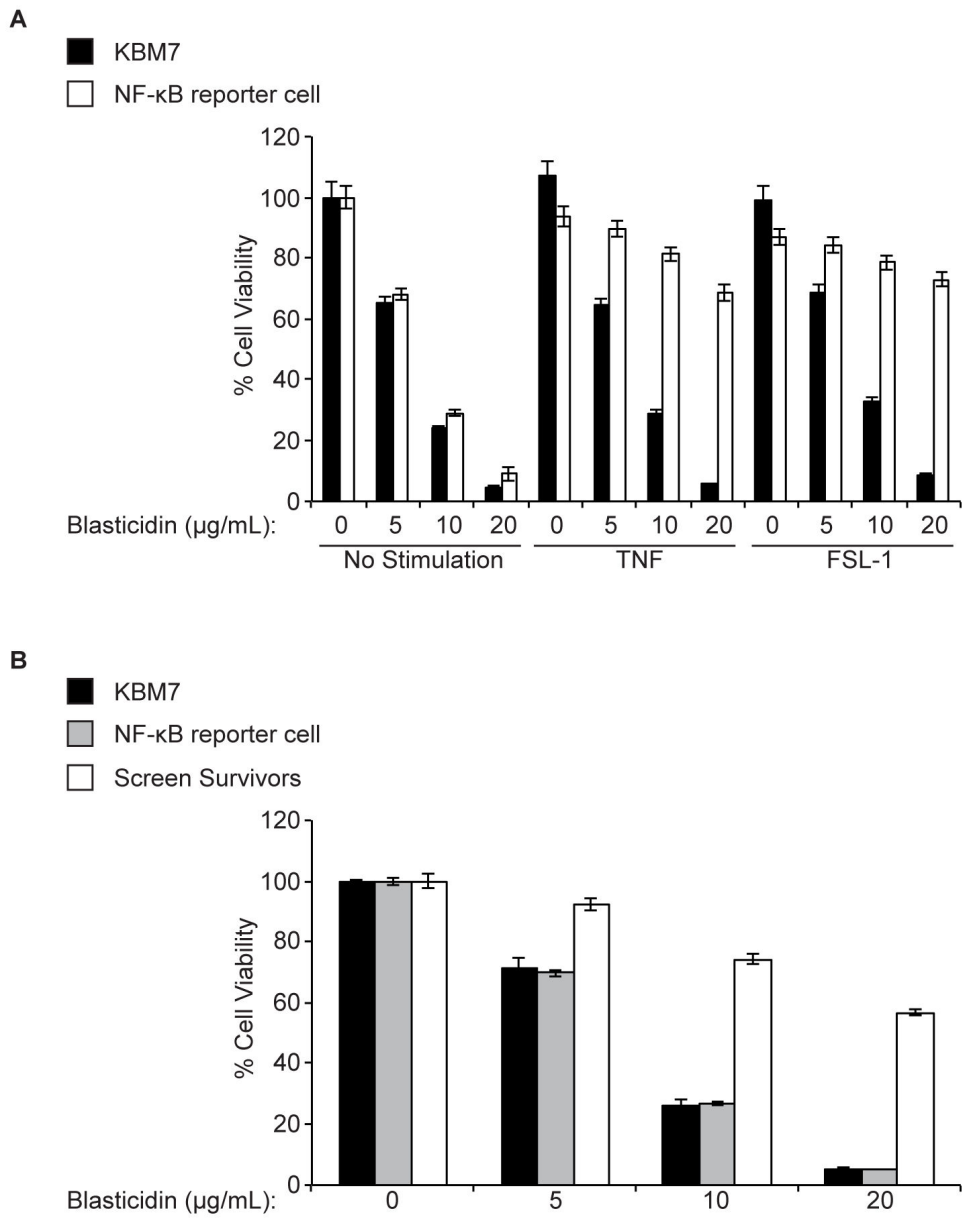
Demonstration of NF-κB reporter function. **A**. Wild-type KBM7 and the clonal NF-κB reporter cell line were treated with varying concentrations of blasticidin in the absence or presence of NF-κB activators (TNF, FSL-1). **B**. Wild-type KBM7, the clonal NF-κB reporter cell line, and the polyclonal screen survivor population were treated with varying concentrations of blasticidin. Cell viability was determined after 24 hours of treatment using the CellTiter Glo assay and results are plotted as percent viability of treated cells compared with untreated cells. Results are mean ± SEM of triplicates and are representative of 3 independent experiments.

**Figure 4:**
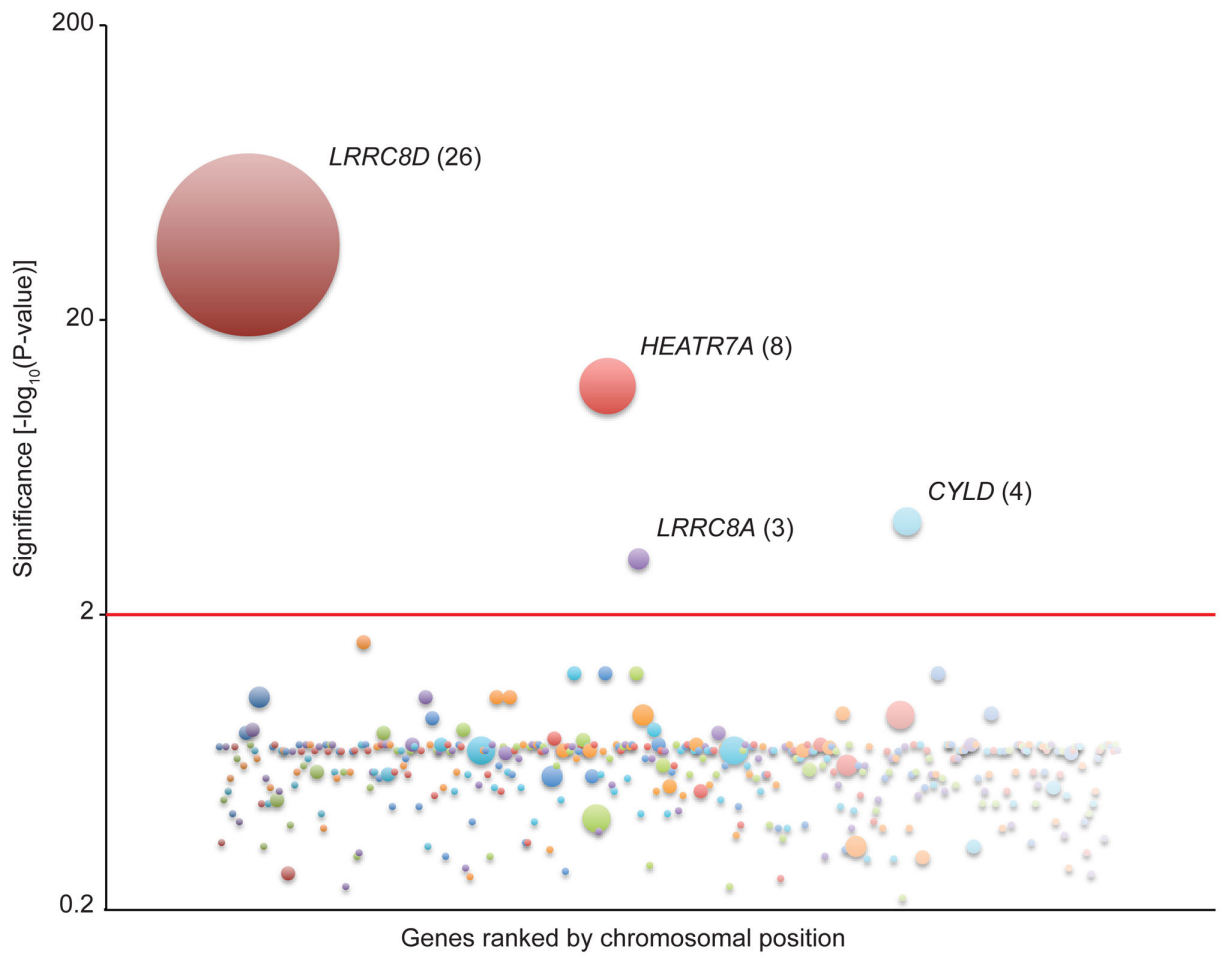
Haploid reporter screen for constitutive inhibitors of NF-κB identifies CYLD. Genes with sequenced inactivating mutations are depicted as circles, the size of which corresponds to the number of independent insertions. Genes are ranked on the x-axis according to their chromosomal position and along the y-axis according to the significance of the enrichment of gene-trap insertions in the indicated gene compared to an unselected control dataset. Genes with a p-value lower than 0.01 are labeled and the number of independent inactivating mutations is indicated between brackets.

**Figure 5:**
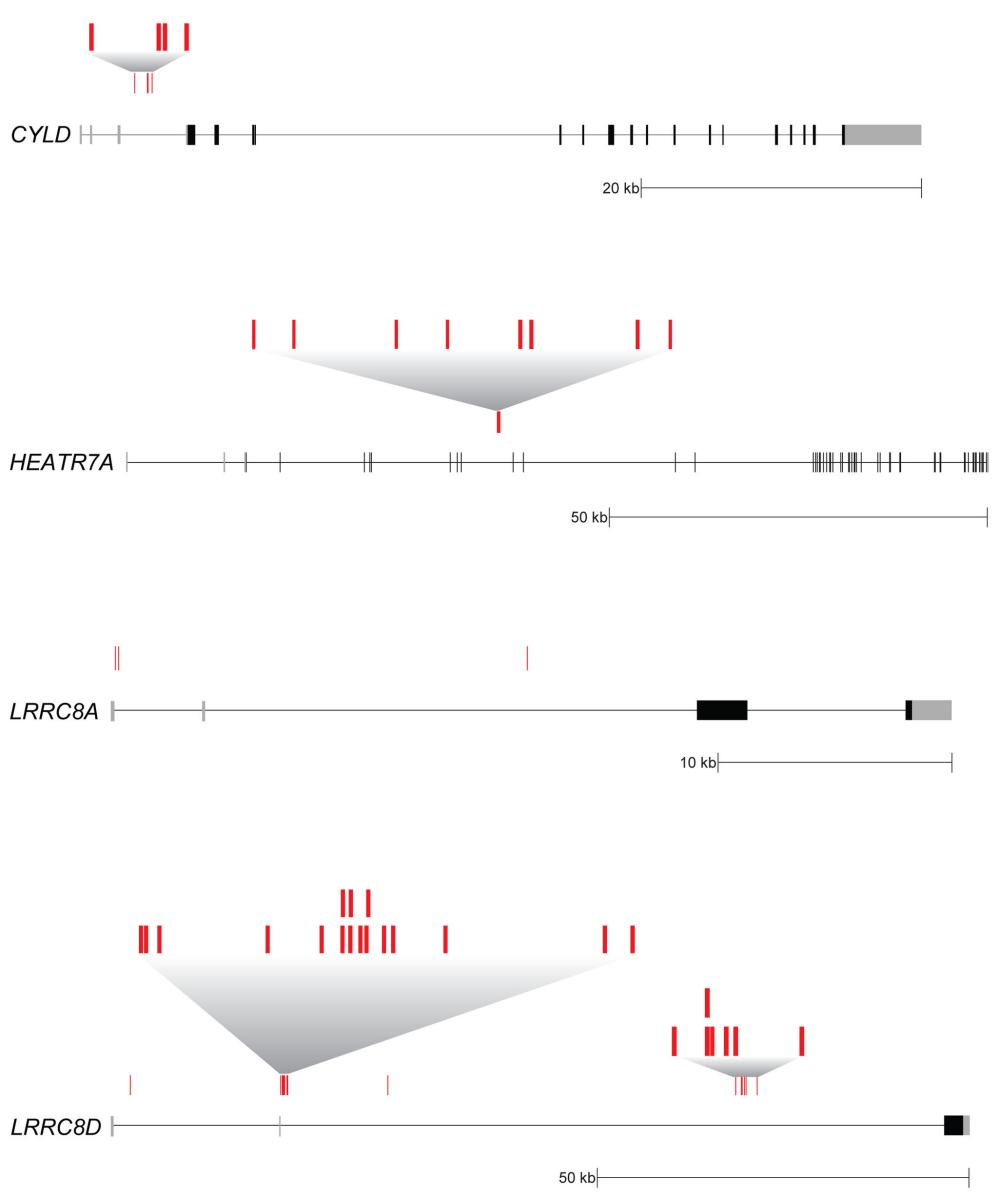
Schematic diagram of inactivating gene-trap insertion sites. The loci of *CYLD* (NM_015247.2), *HEATR7A* (NM_032450.2), *LRRC8A* (NM_019594.3), and *LRRC8D* (NM_001134479.1) are depicted with unique inactivating gene-trap insertion sites (sense orientation or present in an exon) shown in red. For regions where there were many unique insertion sites, the areas are blown up to reveal all unique sites. Gray boxes denote the 5’ and 3’ untranslated regions, and black boxes denote coding exons.

All screens in human haploid cells performed to date have relied on intrinsic phenotypes, such as sensitivity to toxins or protein surface expression, both of which can be easily observed at a cellular level. To provide a clear phenotypic readout for abrogation of NF-κB inhibitor function -and thus improper activation of NF-κB-we generated a NF-κB reporter cell line (Figure 1). We transduced KBM7 cells, which are haploid for all chromosomes but chromosome 8, with a reporter construct that contains a NF-κB transcriptional response element (TRE) and a minimum cytomegalovirus (mCMV) promoter upstream of the blasticidin S resistance gene (*BSR*) from *Bacillus cereus*. Thus, insertional inactivation of genes that normally repress activation of NF-κB would render the reporter cells resistant to blasticidin and provide an easy means to distinguish them from wild-type cells. To ensure that the selected clonal reporter cell line had intact NF-κB regulation, we stimulated both KBM7 cells and the NF-κB reporter cell line with TNF (Figure 2). We saw that both cells displayed similar IκBα degradation kinetics. The selected clonal reporter cell line survived in the presence of blasticidin only when stimulated with NF-κB activators, demonstrating that the reporter functioned properly (Figure 3A). The NF-κB reporter cell line was then mutagenized with a retroviral gene-trap vector, using an established protocol that generally yields a library containing mutations in approximately 98% of genes expressed in KBM7 cells [1]. Mutagenized NF-κB reporter cells were exposed to blasticidin and the survivors were pooled and expanded. The selected mutant population was markedly more resistant to blasticidin than the parental reporter cell line and wild-type KBM7 cells in the absence of any stimulus, suggesting that the survivors contain mutations that cause constitutive activation of NF-κB (Figure 3B). To identify the mutations in the selected mutant population, genomic DNA was harvested from the survivors. The DNA sequences that flank gene-trap insertion sites were amplified, sequenced in parallel, and mapped to the human genome. We identified four genes significantly enriched (p-value < 0.01) for disruptive mutations in our blasticidin-selected population, as compared to a control population of unselected mutagenized cells (Figure 4). In the blasticidin-resistant population, *CYLD*, *HEATR7A*, *LRRC8A*, and *LRRC8D* were represented with 4, 8, 3, and 26 independent inactivating gene-trap insertions (sense orientation or present in an exon), respectively (respective p-values of 6.91 x 10^-5^, 1.09 x 10^-12^, 7.88 x 10^-4^, and 9.71 x 10^-37^) (Figures 4 and 5).

**Figure 6:**
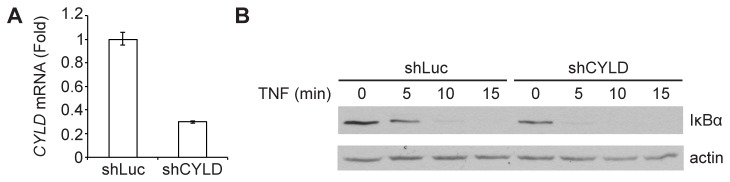
CYLD is constitutively required for normal NF-κB function. **A**. NF-κB reporter cells were transduced with a control hairpin against luciferase (shLuc) or a hairpin against *CYLD* (shCYLD). *CYLD* mRNA levels (mean of triplicates) were determined by quantitative real-time PCR. **B**. NF-κB reporter cells expressing either shLuc or shCYLD were stimulated with 10 ng/mL TNF for various time points. Lysates were analyzed by immunoblot with anti-IκBα. After stripping, the membrane was reprobed with anti-actin antibody to provide a loading control. Data are representative of three independent experiments.

*CYLD* encodes a deubiquitylase (DUB) that targets NF-κB signaling factors and is known to negatively regulate NF-κB activation [12–14]. CYLD is expressed and active at steady-state and it is thought to be constitutively required to prevent spontaneous ubiquitylation of its targets and inappropriate activation of NF-κB in the absence of stimulus [15]. To confirm that CYLD is constitutively required for proper regulation of NF-κB in KBM7 cells, we employed shRNA-mediated knockdown of *CYLD* in NF-κB reporter cells (Figure 6A). In the absence of any stimulus, steady-state IκBα levels are lower in CYLD-depleted cells as compared to cells expressing a control hairpin against luciferase (Figure 6B). Upon TNF stimulation, IκBα expression is lost more rapidly in CYLD-depleted cells. The ability of our screen to specifically identify CYLD, but not other established NF-κB inhibitors that are not required in the absence of stimulus, validates the use of haploid reporter screens to identify particular components of signaling pathways.

*HEATR7A* is predicted to encode a protein of 1,641 amino acids and has no known function. It contains seven HEAT (**H**untington, **e**longation Factor 3, protein phosphatase 2A, **T**OR1) domains, a protein fold found in a variety of proteins including the four that its name derives from. *HEATR7A* is located on chromosome 8, the only chromosome to be present in two copies in KBM7 cells suggesting that the mutations caused by the gene trap insertions in *HEATR7A* are of a dominant nature.

LRRC8A and LRRC8D represent two of five members of the leucine-rich repeat-containing 8 (LRRC8) family of proteins that are composed of four transmembrane domains at the N-terminus, followed by up to 17 leucine-rich repeats [16]. Their function is poorly understood, but LRRC8A and LRRC8C have been implicated in B cell development and adipocyte differentiation, respectively [17–19]. Thus it is not inconceivable that there may exist a link between LRRC8s and NF-κB, given the established function of NF-κB in lymphocyte differentiation and its emerging role in metabolic disorders [11,20,21]. Given that LRRC8D was identified with high confidence in our screen, we sought to examine its function by isolating two clones that carry gene trap insertions in the *LRRC8D* gene. While the LRRC8D mutant cell lines were notably resistant to blasticidin, examination by immunoblotting revealed no obvious impact of LRRC8D deficiency on IκBα and p100 degradation (data not shown). Our attempts to demonstrate a possible effect of the LRRC8D disruptions on blasticidin import as an explanation for the observed level of resistance were inconclusive, as the fluorescently labeled or biotinylated versions of blasticidin we prepared were themselves inactive on KBM7 cells. Thus we can neither exclude the possibility that resistance is conferred by differences in intracellular blasticidin levels, nor discount the possibility of alternative modes of NF-κB activation not accounted for by degradation of IκBα and p100 [22].

## Discussion

Most genetic screens performed in human haploid cells have sought to identify components in pathways required for cell death in response to a lethal insult. Here we have demonstrated that KBM7 cells can be modified with genetically encoded transcriptional reporters to study more diverse cellular processes. While we chose to screen for regulators -specifically, inhibitors-of NF-κB, our method could presumably be applied to study the approximately 1,391 human sequence specific DNA binding transcription factors, many of whose binding site profiles have recently been described [9,23].

By using resistance to blasticidin as our reporter read-out, we were able to perform a selection -a genetic screen where only mutants of interest survive-to identify mutants that constitutively turned on the reporter. In principle, one could perform a screen in a reversed fashion, in which only mutants that fail to turn on the reporter survive, for example by exploiting thymidine kinase or some other protein whose expression could induce cell death. Identification of positive regulators of transcription factors should thus be possible. Fluorescent reporters could likewise be used in screens for both positive and negative transcriptional regulators. Since stringency in this type of screen would be more adjustable than in a lethal screen, mutations that result in intermediate phenotypes might be more easily recovered.

While the identification of CYLD validated our approach, we were unable to identify mutations in other known negative regulators of NF-κB. Perhaps the selection we performed was particularly stringent. Presumably, only mutations that resulted in constitutive activation of NF-κB could be recovered. Thus, inhibitors whose function or expression is induced by NF-κB in a negative feedback loop, such as A20 and Cezanne, may not meet that criterion. In addition, loss-of-function mutations in dominant-negative adaptors such as MyD88s, IRAK-M, and SARM would not result in constitutive activation of NF-κB. We did not recover mutations in IκBs, possibly because there is some redundancy in function in KBM7 cells and removal of just one IκB is not sufficient for constitutive activation of NF-κB. In contrast, CYLD qualifies as a constitutively active inhibitor that prevents spontaneous ubiquitylation of its targets [15]. CYLD mutations are associated with constitutive activation of NF-κB in multiple myeloma cells and B cells from mice deficient for wild-type CYLD exhibited constitutive activation of NF-κB [15,24,25].

Our haploid reporter screen confirms the absolute requirement of CYLD function for proper regulation of NF-κB and further supports constitutive NF-κB activity as the mechanism underlying the development of human diseases associated with CYLD mutations. Our screen identified genes not previously known to be involved with NF-κB regulation. Their exact role remains to be determined. The ability to perform haploid reporter screens in human cultured cells opens up many new cellular processes for investigation.

## Materials and Methods

### Plasmids

The NF-κB reporter was created by digesting pTRH1-NF-κB-dscGFP (System Biosciences) with SpeI and SalI to remove the dscGFP. The same restriction sites were used to insert a synthesized DNA fragment encoding mCMV-NheI-Kozak sequence-BamHI-*Bacillus cereus BSR*-Thosea asigna virus 2A sequence-XbaI-Rabbit *CYP4B1*. Lentiviral shRNAs were obtained from The RNAi Consortium (TRC) collection of the Broad Institute. The TRC numbers for the shRNAs used are TRCN0000072245 (shLuc) and TRCN0000039632 (shCYLD).

### Cells

KBM7 cells were grown in Iscove’s modified Dulbecco’s medium (IMDM) with 10% heat-inactivated fetal serum (IFS) [3,26]. A NF-κB reporter cell line was created by transducing KBM7 cells with the NF-κB reporter construct described above and single cells were sorted into individual wells of 96 well plates. A clone that remained haploid and that died in the presence of blasticidin S (Invivogen), but survived in blasticidin when stimulated with NF-κB agonists was selected for the screen.

### Reporter haploid genetic screen

The screening procedure has been described previously [1–6]. Briefly, 100 million NF-κB reporter cells were infected with gene-trap retrovirus to create a mutagenized library. 200 million mutagenized cells were then plated 100,000 cells/well in 96 well plates with half in 10 µg/mL blasticidin S (Invivogen) and half in 15 µg/mL blasticidin S for 6 days. After 6 days, the media above the cells was replaced with antibiotic-free IMDM. Survivors were allowed to expand for about three additional weeks before harvest. DNA was harvested from ~30 million cells from each of the two blasticidin conditions.

### Sequence analysis of gene-trap insertion sites

The mapping of the insertion sites was done as previously described [1–6]. In short, DNA sequences flanking gene-trap insertions sites were amplified using an inverse PCR protocol followed by sequencing using the Genome Analyzer platform (Illumina). The sequences were then aligned to the human genome. The number of inactivating mutations (that is, sense orientation or present in exon) per individual gene was counted as well as the total number of inactivating insertions for all genes. Enrichment of a gene in the screen was calculated by comparing how often that gene was mutated in the screen compared to how often the gene carries an insertion in the control data set. For each gene, a P-value (corrected for false discovery rate) was calculated using the one-sided Fisher exact test.

### Immunoblot analysis of TNF stimulated cells

Two million cells were used per condition. Cells were washed once in ice-cold PBS and then lysed in buffer containing 50 mM Tris-HCl pH 7.4, 150 mM NaCl, 0.5 mM EDTA, protease inhibitors (Roche) and 1% (v/v) NP40. Protein concentrations of lysates were determined by Bio-Rad Protein Assay and then normalized across samples. Proteins from total lysates were resolved by 10% SDS-PAGE and analyzed by immunoblotting with primary antibodies: mouse anti-IκBα (BD #610690) at 1:500 dilution and mouse anti-actin (BD #612656) at 1:10,000. Horseradish peroxidase (HRP)-conjugated sheep anti-mouse IgG (GE NXA931) was used at 1:5,000 dilution. Restore PLUS Western Blot Stripping Buffer (Thermo) was used to strip the membranes between probing for IκBα and actin.

### Cell viability assay

CellTiter-Glo Luminescent Cell Viability Assay (Promega) was used to quantify cell viability. 200,000 cells were seeded per well in Optilux clear-bottom 96-well plates (BD Falcon) in IMDM or IMDM supplemented with varying concentrations of Blasticidin S (Invivogen), TNF-α (Invivogen), FSL-1 (Invivogen) for 24 hours before reading on a Luminoskan Ascent luminometer (Thermo Scientific).

### Quantitative real-time PCR

RNA was extracted using a RNAeasy kit (QIAGEN) followed by on-column DNase I (QIAGEN) digestion. SuperScript III First-Strand Synthesis System (Invitrogen) was used for the reverse transcription reaction using Oligo dT primers. SYBR Green PCR Master Mix (Applied Biosystems) was used according to manufacturer’s instructions. Real-time PCR reactions were run on an ABI 7900HT machine. PCR volume was 20 µL (96-well plate), and data values were derived from three replicates using the comparative Ct method. Primers used for *CYLD* were 5’ GGTAATCCGTTGGATCGGTCAGC 3’ and 5’ TGCAAACCTAGAGTCAGGCCTGC 3’. Primers used for *GAPDH* were 5’ ACCCACTCCTCCACCTTTGACG 3’ and 5’ CACCCTGTTGCTGTAGCCAAATTCG 3’.
